# Supplementary material for: A Critical Appraisal of National and International Clinical Practice Guidelines Reporting Nutritional Recommendations for Age-Related Macular Degeneration: Are Recommendations Evidence-Based?
Source: Nutrients. 2019 Apr 11;11(4):823. doi: 10.3390/nu11040823 (PMC6520821; doi:10.3390/nu11040823)
Supplement: Supplementary file 1 [file nutrients-11-00823-s001.pdf]

Supplementary material

Search Strategy

Database: Embase <1996 to 2019 Week 03>, Ovid MEDLINE(R) <2014 to January Week 3 2019>
----

1 exp clinical pathway/
2 exp clinical protocol/
3 exp consensus/
4 exp consensus development conference/
5 exp consensus development conferences as topic/
6 exp critical pathways/
7 exp guideline/
8 exp guidelines as topic/
9 exp practice guideline/
10 exp practice guidelines as topic/
11 exp health planning guidelines/
12 (guideline or practice guideline or consensus development conference or consensus development conference, NIH).pt.
13 (position statement* or policy statement* or practice parameter* or best practice*).ti,ab,kf,kw.
14 (standards or guideline or guidelines).ti,kf,kw.
15 ((practice or treatment* or clinical) adj guideline*).ab.
16 (CPG or CPGs).ti.
17 consensus*.ti,kf,kw.
18 consensus*.ab. /freq=2
19 ((critical or clinical or practice) adj2 (path or paths or pathway or pathways or protocol*)).ti,ab,kf,kw.
20 recommendat*.ti,kf,kw.
21 (care adj2 (standard or path or paths or pathway or pathways or map or maps or plan or plans)).ti,ab,kf,kw.
22 (algorithm* adj2 (screening or examination or test or tested or testing or assessment* or diagnosis or diagnoses or diagnosed or diagnosing)).ti,ab,kf,kw.
23 (algorithm* adj2 (pharmacotherap* or chemotherap* or chemotreatment* or therap* or treatment* or intervention*)).ti,ab,kf,kw.
24 or/1-23
25 ((macul* or retina* or choroid*:TI) and (degener* or neovasc*:TI)).mp. [mp=ti, ab, hw, tn, ot, dm, mf, dv, kw, fx, dq, nm, kf, px, rx, an, ui, sy]
26 ((macul* or retina* or choroid*:AB) and (degener* or neovasc*:AB)).mp. [mp=ti, ab, hw, tn, ot, dm, mf, dv, kw, fx, dq, nm, kf, px, rx, an, ui, sy]
27 or/25-26
28 24 and 27
29 limit 28 to last 10 years

****
